# TriticeaeSSRdb: a comprehensive database of simple sequence repeats in *Triticeae*

**DOI:** 10.3389/fpls.2024.1412953

**Published:** 2024-05-22

**Authors:** Tingting Li, Shaoshuai Cai, Zhibo Cai, Yi Fu, Wenqiang Liu, Xiangdong Zhu, Chongde Lai, Licao Cui, Wenqiu Pan, Yihan Li

**Affiliations:** ^1^ College of Bioscience and Engineering, Jiangxi Agricultural University, Nanchang, Jiangxi, China; ^2^ State Key Laboratory for Crop Stress Resistance and High-Efficiency Production, Northwest A&F University, Yangling, Shaanxi, China; ^3^ The Public Instrument Platform of Jiangxi Agricultural University, Jiangxi Agricultural University, Nanchang, Jiangxi, China

**Keywords:** genome, microsatellite, SSR, database, molecular breeding

## Abstract

Microsatellites, known as simple sequence repeats (SSRs), are short tandem repeats of 1 to 6 nucleotide motifs found in all genomes, particularly eukaryotes. They are widely used as co-dominant markers in genetic analyses and molecular breeding. *Triticeae*, a tribe of grasses, includes major cereal crops such as bread wheat, barley, and rye, as well as abundant forage and lawn grasses, playing a crucial role in global food production and agriculture. To enhance genetic work and expedite the improvement of *Triticeae* crops, we have developed TriticeaeSSRdb, an integrated and user-friendly database. It contains 3,891,705 SSRs from 21 species and offers browsing options based on genomic regions, chromosomes, motif types, and repeat motif sequences. Advanced search functions allow personalized searches based on chromosome location and length of SSR. Users can also explore the genes associated with SSRs, design customized primer pairs for PCR validation, and utilize practical tools for whole-genome browsing, sequence alignment, and *in silico* SSR prediction from local sequences. We continually update TriticeaeSSRdb with additional species and practical utilities. We anticipate that this database will greatly facilitate trait genetic analyses and enhance molecular breeding strategies for *Triticeae* crops. Researchers can freely access the database at http://triticeaessrdb.com/.

## 1 Introduction

The *Triticeae* tribe, a member of the *Poaceae* grass family’s subfamily *Pooideae*, encompasses a total of 503 diploid and polyploid species distributed among 27 genera (Soreng et al., 2015). This tribe includes economically significant cereal crops, such as bread wheat (*Triticum aestivum*), barley (*Hordeum vulgare*), and rye (*Secale cereale*), along with their wild relatives (Sato et al., 2021). Since the Neolithic Agricultural Revolution, these crops have played essential roles in human sustenance and global food security (Li et al., 2023).

Recent advancements in novel genome assembly algorithms and strategies have progressively improved the quality of genome assemblies in *Triticeae*, resulting in a rapid increase in the availability of assemblies (Adamski et al., 2020). These high-quality assemblies enable the decoding of chromosome and gene evolutionary history through clade-specific comparative genomic studies (Chen et al., 2020). Deciphering the genomes of *Triticeae* species has revealed their characteristic features of large, complex, and highly repetitive genomes. For instance, diploid *Triticeae* species like *H. vulgare* and *S. cereale* have genome sizes ranging from 4.3 Gb to 7.9 Gb. The tetraploid *T. dicoccoides* genome comprises 10.5 Gb of genomic sequence, while the hexaploid *T. aestivum* Chinese Spring genome spans 14.5 Gb, with approximately 80% of these genomes consisting of repetitive elements (Wang et al., 2022). In addition to the assembly of high-quality reference genomes, recent *Triticeae* pan-genome studies have commenced, capturing the genomic diversities of these species and enhancing our understanding of the genetic basis of domestication and environmental adaptation in *Triticeae* crops (Gao et al., 2023). However, these genomic complexities present challenges for genetic studies and the identification of agronomic traits in *Triticeae* crops.

The majority of *Triticeae* breeding programs heavily rely on conventional breeding selection, which involves replicated and time-consuming field trials. However, Marker-assisted selection (MAS) has emerged as an increasingly popular and valuable tool for tracking alleles associated with key agricultural traits (Hasan et al., 2021). Recent advancements in the availability of reference sequences and high-throughput genotyping platforms for major *Triticeae* species have facilitated comprehensive genome analysis. This has provided valuable insights into marker development and laid the foundation for identifying DNA markers closely linked to target traits. In various crops, microsatellites, or simple sequence repeat (SSRs) have become the preferred marker type for MAS, further enhancing its effectiveness and application (Duhan and Kaundal, 2021).

SSRs are short nucleotide motifs that are tandemly repeated and range in length from 1 to 6 base pairs (Tóth et al., 2000). The high polymorphism of microsatellites arises from the variation in the number of repeat units among different alleles. SSR markers offer several advantages, including easy detection through polymerase chain reaction (PCR), unambiguous scoring, and high reproducibility (Powell et al., 1996). Consequently, they have been extensively employed in genetic studies, encompassing genetic diversity assessment, phylogenetic analysis, genetic linkage mapping, and quantitative trait locus (QTL) mapping (Kalia et al., 2011). The identification and development of SSR markers at a genome-wide scale have been greatly facilitated by the availability of reference sequences and high-throughput genotyping platforms. Microsatellites serve significant roles in genome organization and are widely recognized as popular neutral genetic markers (Schlötterer, 2000). The characterization and application of SSR markers hold practical implications for breeding programs, providing valuable assistance to breeders in genotype association and selection processes. Despite the impact of the rise of high-throughput sequencing technologies on traditional molecular markers, SSR technology, which relies on PCR systems, can be conducted in any molecular biology laboratory, and has an absolute advantage, particularly in the rapid preliminary screening of materials. In contrast, high-throughput sequencing technologies are limited by the cost of instruments and are not widely applicable in laboratories. As a result, successful whole-genome identification and development of SSR markers have been achieved in various species (Deng et al., 2016; Guan et al., 2019; Lu et al., 2019). To facilitate data sharing, user-friendly and accessible databases have been established, such as LegumeSSRdb (Duhan and Kaundal, 2021), citSATdb (Duhan et al., 2020), PSSRD (Song et al., 2021), and MSDB (Avvaru et al., 2017, 2020).

Given the various applications of SSRs in genetic and genomic research, such as genetic mapping, diversity analysis, marker-assisted selection, and population genetics, it is evident that there is a lack of a comprehensive database specifically catering to *Triticeae* species. Therefore, in addition to the existing databases, the development of a dedicated database that focuses on the genomic data of the *Triticeae* tribe and incorporates user-friendly features would be a valuable genomic resource for improving and characterizing *Triticeae* crops. In this study, we introduce TriticeaeSSRdb, an integrated web resource that offers a wide range of services, including JBrowse visualization, Basic Local Alignment Search Tool (BLAST), *in silico* SSR prediction, primer design for genotyping, and other enriching features. The species included in our database have undergone complete genome sequencing, ensuring a more comprehensive representation of SSR information. With the establishment of TriticeaeSSRdb, we aim to not only provide comprehensive and representative SSR information but also enable large-scale systematic and comparative analyses of SSRs in *Triticeae* species.

## 2 Materials and methods

### 2.1 Data collection

A total of 21 species from the *Triticeae* tribe were included in our study (**Supplementary Table S1**). Sequence information was downloaded in FASTA format, and the genomic annotations were obtained in GFF or GTF format. If multiple versions were available for the same species, we selected the most recent or favored assembly for download. The genomic information of six species, namely *Aegilops tauschii*, *Hordeum vulgare*, *T. aestivum*, *T. dicoccoides*, *T. spelta*, and *T durum* was obtained from the Ensembl Plants database (https://plants.ensembl.org/). The genomes of nine species, including *Ae. bicornis*, *Ae. longissima*, *Ae. searsii*, *Ae. sharonensis*, *Ae. speltoides*, *Ae. umbellulata*, *T. aestivum* ssp. *tibetanum* Shao, *T. monococcum*, and *T. urartu*, were downloaded from the National Center for Biotechnology Information (NCBI) Database (https://www.ncbi.nlm.nih.gov/). *Dasypyrum villosum*, *H. marinum*, *Leymus chinensis*, and *Thinopyrum elongatum* (4 species) were collected from the China National Center for Bioinformation - National Genomics Data Center (CNCB-NGDC) (https://ngdc.cncb.ac.cn/). *Hordeum vulgare* var. *nudum* was obtained from the WheatOmics database (http://wheatomics.sdau.edu.cn/jbrowse.html). *H. spontaneum* was retrieved from the IPK database (https://barley-pangenome.ipk-gatersleben.de/).

### 2.2 SSR identification and primer design

The genome sequences of all 21 species were analyzed using Pytrf v1.3.0, a Python package specifically designed for detecting both exact or perfect SSRs in genomic sequences (https://github.com/lmdu/pytrf/blob/master/docs/index.rst) (Du et al., 2018). Microsatellites were identified based on specific criteria for six different categories: mono-nucleotide repeats (MNRs) with a minimum repeat count of 15, di-nucleotide repeats (DNRs) with a minimum repeat count of 10, tri-nucleotide (TNRs) repeats with a minimum repeat count of 8, and tetra- (TtNRs), penta- (PNRs), and hexa-nucleotide repeats (HNRs) with a minimum repeat count of 5. To facilitate primer design, an in-house Python script was developed to exclude a total of 500 bp flanking sequences upstream (250 bp) and downstream (250 bp) of the identified SSR regions. The forward and reverse primers flanking the microsatellite repeat motifs were designed in batches using the primer3_core program v2.6.1 (Untergasser et al., 2012). Two Perl scripts, p3_in.pl and p3_out.pl, were implemented to enable seamless data interchange between Pytrf and the primer modeling software Primer v3.0. The primer design parameters were set as follows: primer size ranging from 20 to 22 base pairs, GC content between 40% and 60%, melting temperature between 55°C and 65°C, and expected product size ranging from 100 to 500 base pairs.

### 2.3 Functional annotation of SSR markers

To determine the precise genomic locations of the identified SSRs, we developed a custom Python script. Using the available genomic annotation file, we categorized the SSRs into intergenic and genic regions. The genic regions were further subdivided into intron, untranslated region (UTR), and coding sequence (CDS). The genomic distribution of SSRs was visualized using the RIdeogram v0.2.2 R package (Hao et al., 2020). To facilitate data visualization, we integrated comprehensive gene function annotations for all 21 *Triticeae* species into the Jbrowse genome browser v1.16.11 within the TriticeaeSSRdb database (Buels et al., 2016). This genome browser allows for the visualization of markers in relation to the reference sequence, gene coordinates, and detailed structural and functional information. This integration enhances the accessibility and understanding of the SSR data within the context of gene features and functions. Gene ontology (GO) enrichment analyses were conducted using KOBAS v3.0 (http://bioinfo.org/kobas).

### 2.4 Database implementation and web interface

The main website of the database is hosted on a Tencent Cloud server with specifications of an 8-core CPU, 64GB RAM, 500GB storage, and a 10Mbps bandwidth (https://cloud.tencent.com/). The domain name (http://triticeaessrdb.com/) was registered and linked to the server’s IP address. The server operates on the Linux CentOS v7.9 operating system (http://www.centos.org). To facilitate platform development and web page creation, a Nginx v1.25.0 reverse proxy server architecture was configured.

**Figure 1:**
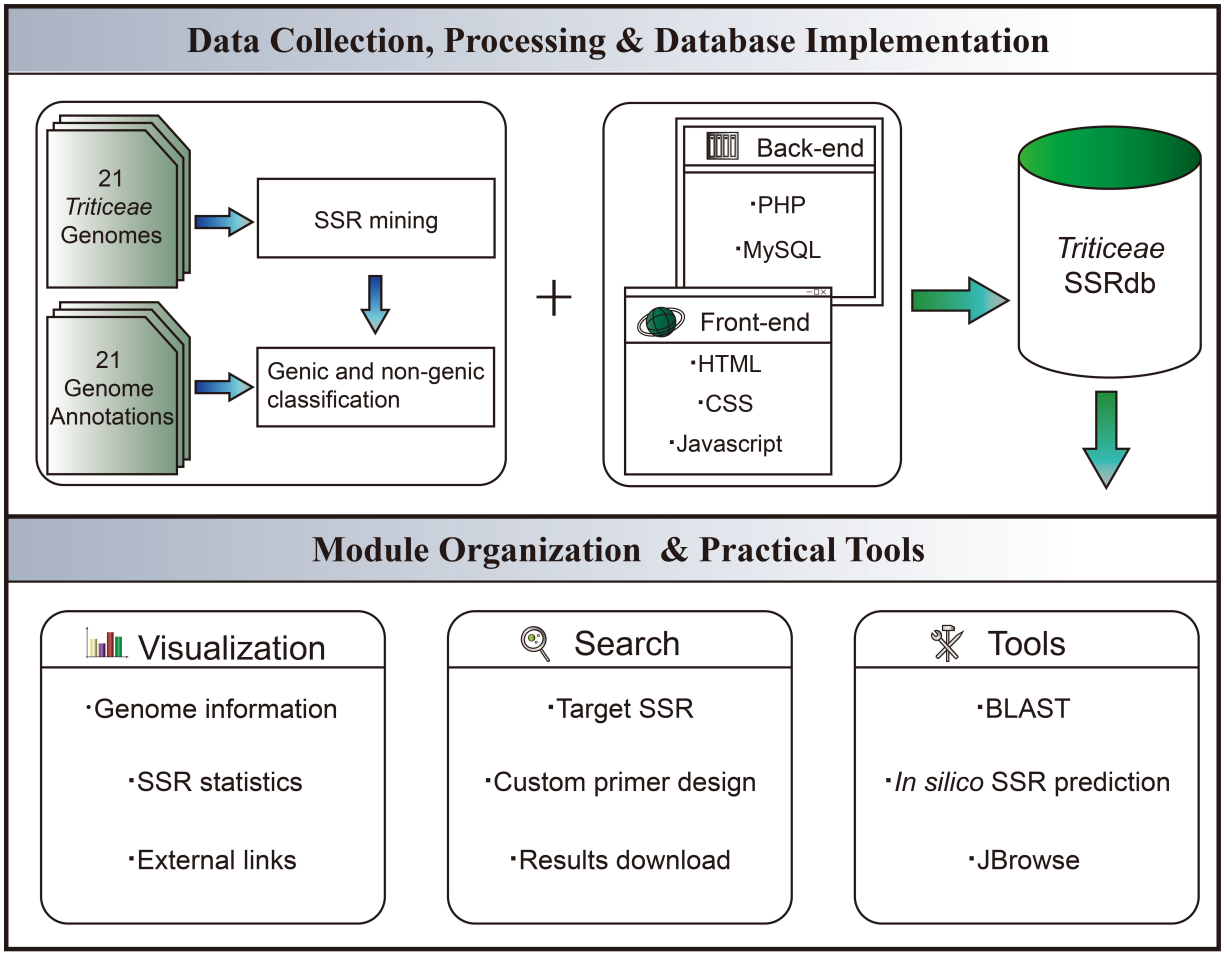
Schematic Workflow of TriticeaeSSRdb.

The front-end web interface was developed using HTML5 (https://www.w3.org/html/), JavaScript (https://www.javascript.com/), CSS3 (http://www.w3.org), and Bootstrap5 (https://getbootstrap.com/). On the server side, the back-end was implemented using PHP (https://www.php.net/). MySQL v5.7.22 (https://www.mysql.com/) was utilized for storing, maintaining, and operating the microsatellite information. Custom PHP code was written to facilitate data retrieval from MySQL, which was then transferred to the front end for display. Data visualization charts were implemented using the Chart.js (https://www.chartjs.org/) component. Real-time primer designing functionality was achieved by integrating Primer3 into TriticeaeSSRdb. Furthermore, we employed a back-end Perl script called Microsatellite Identification Tool (MISA) (Thiel et al., 2003) and a Python script named Pytrf to facilitate the *in silico* prediction of microsatellites within uploaded sequences. For sequence homology searches, the BLAST was implemented using ViroBlast, a standalone BLAST web server. To enable online BLAST searches, ViroBLAST constructed a standalone database specifically for the 21 *Triticeae* species (Deng et al., 2007). Local scripts were also modified to enhance search services. The whole workflow of TriticeaeSSRdb is illustrated in **Figure 1**.

## 3 Results

### 3.1 SSR abundance and distribution in *Triticeae* species

In the current study, we characterized and compared the distribution of SSRs in 21 *Triticeae* genomes. A total of 3,891,705 microsatellites were identified, with an overall frequency of 27.21 SSRs/Mb (**Supplementary Table S1**). Considering their hexaploid genome characteristics, *T. aestivum* ssp. *tibetanu* Shao (321,257), *T. spelta* (314,128), and *T. aestivum* (269,000) exhibited a relatively higher total number of SSRs. At the diploid level, *H. vulgare* had the highest number of SSRs (304,626), while *Ae. tauschii* (101,264) and *H. marinum* (71,983) had the fewest SSRs. The maximum microsatellite density was observed in species *H. vulgare* (72.09 SSRs/Mb), whereas the minimum was observed in species *T. aestivum* (18.49 SSRs/Mb). Generally, species with larger genomes exhibited lower SSR density. Previous studies have reported a significantly negative correlation between SSR frequency and genome sizes in *Poaceae* species (Deng et al., 2016). However, our analysis of the 21 *Triticeae* species did not yield similar results. Although differences in genome size may contribute to the level of microsatellite repetition, we found no evidence of a relationship between SSR density and genome size (Portis et al., 2018).

### 3.2 Characterization of SSR motifs

The predicted SSR loci were classified into six categories: MNR, DNR, TNR, TtNR, PNR, and HNR. Across all species, MNR, DNR, and TNR accounted for 90.06% of the total SSR. DNR was the most abundant category (49.53%), followed by TNR and MNR (**Supplementary Table S2**). Among the mono-nucleotide repeats, *Th. elongatum* had the highest percentage, while *T. dicoccoides* and *T. durum* had the lowest. Dimeric repeats had the highest occurrence in most species. Regarding trimeric repeats, three species from the *Hordeum* genus (*H*. *vulgare* var. *nudum*, *H*. *vulgare*, and *H*. *spontaneum*) and *Th*. *elongatum* exhibited higher percentages compared to other *Triticeae* species, suggesting their distinct evolutionary history. In contrast, SSRs with longer repeat motifs demonstrated significantly lower frequency, with average ratios of 7.71%, 0.85%, and 1.38% for tetrameric, pentameric, and hexameric repeats, respectively.

The distribution of microsatellites in *Triticeae* species was examined in terms of repeat unit length. Our analysis revealed a negative correlation between repeat length and frequency, indicating that longer repeats were less common (**Supplementary Figure S1**; **Supplementary Table S3**). Moreover, the abundance of SSRs decreased as the repeat number increased. It is worth noting that the decline rate of mono-, di-, tri-, and tetra-nucleotide repeats was noticeable, while the decline trend of penta- and hexa-nucleotide repeats was slightly less steep.

### 3.3 Analysis of SSR motif sequence composition

A total of 3424 distinct SSR motifs were identified, encompassing all possible base combinations of mono-nucleotide (4 types), di-nucleotide (12 types), tri-nucleotide (60 types), and tetra-nucleotide (240 types) repeats. Additionally, variations of penta- and hexa-nucleotide repeats contributed 955 and 2153 additional motifs, respectively (**Supplementary Table S4**). We further conducted a comprehensive analysis of individual repetitive motifs for each type of SSR (**Supplementary Table S5**). Considering the relatively low GC content observed in *Triticeae* species, the base composition of repeat motifs exhibited a notable bias towards A and T nucleotides. Remarkably, the motif (AT)n ranked among the top five most frequently occurring motifs in the 20 *Triticeae* genomes. Notably, AT repeats emerged as the most frequent motif overall, accounting for 10.11% of all SSRs in *Triticeae*. In contrast, GC repeats were exceptionally rare, with (G)n accounting for 0.157%-18.079%, (C)n accounting for 0.158%-18.245%, (GC)n accounting for 0.003%-0.050%, and (CG)n accounting for 0.001%-0.030%. The most prevalent motifs in *Triticeae* species encompassed mono- to hexanucleotide repeats, including C (40.63% of mono-nucleotide repeats), AT (20.40% of di-nucleotide repeats), AAG (7.44% of tri-nucleotide repeats), TTAA (10.14% of tetra-nucleotide repeats), CCTCT (3.54% of penta-nucleotide repeats), and TTTTCA (6.17% of hexa-nucleotide repeats).

### 3.4 Functional annotation of SSRs

The identified SSR loci were categorized based on their distribution across the pseudomolecules. The distribution of SSR loci was found to be relatively uneven among the different chromosomes (**Supplementary Table S6**). In *H. vulgare*, for example, the density of SSRs varied from 90.91 SSRs/Mb (chr4H) to 53.42 SSRs/Mb (chr5H), with an average density of 68.99 SSRs/Mb. Among the chromosomes, the longest linkage group, chr2H in *H. vulgare*, contained a total of 39,071 SSRs, while the fourth longest chromosome, chr4H, had the highest number of SSRs (55,487). Furthermore, the analysis of chromosome location revealed a tendency for the identified SSRs to be located on the distal chromosome arm rather than in the pericentromeric region (**Supplementary Figure S2**). This may be attributed to the fact that the terminal regions of chromosomes are often gene-rich and contain other functional elements, which are relatively stable and subject to less selective pressure and recombination, thus providing more opportunities for the accumulation of microsatellite repeat sequences.

Based on the corresponding annotation file, we successfully categorized the identified SSRs as genic and intergenic SSRs. Genic SSRs were further classified into intron, UTR, and CDS regions. The majority of SSRs were found in the intergenic region, ranging from 83.8% (*H. spontaneum*) to 58.5% (*Ae. tauschii*), with an average ratio of 69.9% (**Supplementary Table S7**). Noncoding regions exhibited a higher frequency of SSRs compared to CDS, possibly due to negative selection against frameshift mutations in coding regions. Genic SSR markers, in contrast to noncoding microsatellites, are highly portable among related species, making them useful as anchor markers for comparative genetics purposes (Varshney et al., 2005). In terms of the genic region, 19.66% and 8.56% of SSRs were located in the intron and CDS regions, respectively. It is important to note that the available released genomes employ different annotation workflows, and a proportion of genomes lack UTR annotations. With the exception of *Ae*. *tauschii* and *T*. *dicoccoides*, where 10.5% and 7.2% of SSRs were predicted to be in the UTR, respectively, the remaining genomes showed an annotation of 1.1% to 5.3% of SSRs in this region.

Genic SSRs serve as valuable functional markers due to their involvement in gene expression and function in plants (Li et al., 2004). To investigate the potential biological functions of SSR-associated genes, we conducted a GO enrichment analysis using *H. vulgare* as a model. The genic SSRs showed significant enrichment in 232 GO terms (corrected p-value < 0.05), encompassing 127 biological processes, 37 cellular components, and 68 molecular functions (**Supplementary Table S8**). The top three enriched terms in the molecular function category were protein binding (GO:0005515), kinase activity (GO:0016301), and protein serine/threonine kinase activity (GO:0004674), while the most common terms in the cellular components category were plasma membrane (GO:0005886), cytosol (GO:0005829), and nucleus (GO:0005634). These genes were found to be implicated in various biological functions (e.g., GO:0006468, protein phosphorylation; GO:0006952, defense response; and GO:0042742, defense response to bacterium), and demonstrated involvement in response to diverse stresses (e.g., GO:0006979, response to oxidative stress; GO:0009651, response to salt stress; and GO:0071472, cellular response to salt stress) within the biological process category. These results indicate the extensive involvement of SSR tightly linked genes in diverse signal transduction pathways and their ability to adapt to various stresses. Therefore, the development of new SSR markers can contribute to the improvement of genetic analysis, QTL mapping, and molecular breeding in *Triticeae* crops.

### 3.5 Database implementation and practical tools

**Figure 2:**
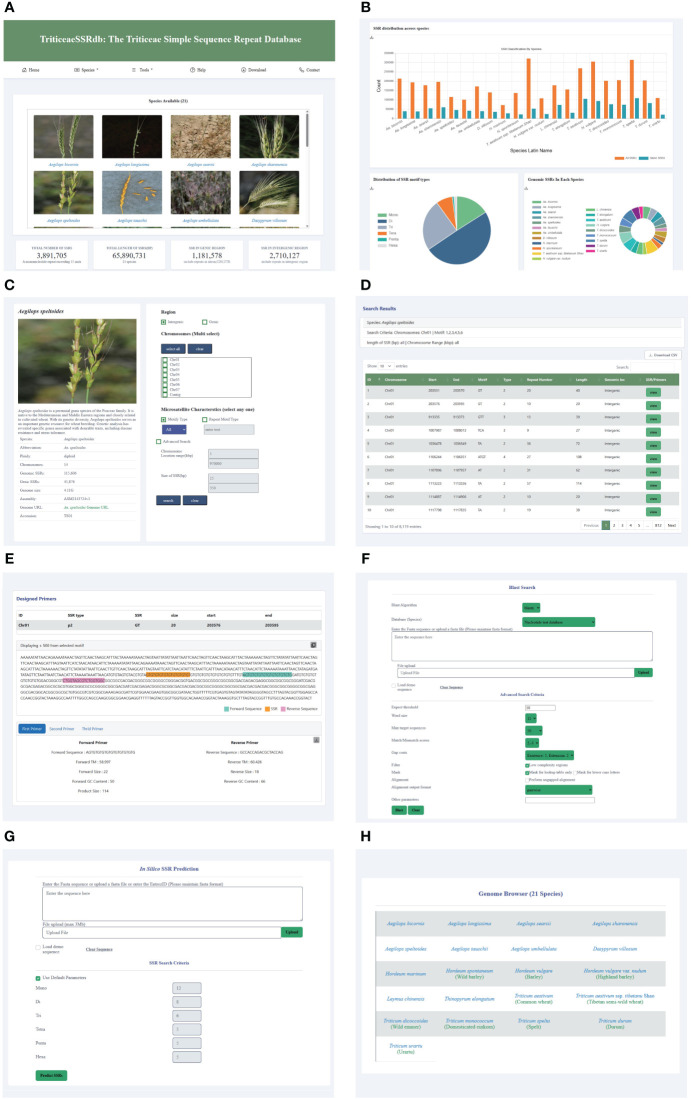
Main Functions and Modules of TriticeaeSSRdb. **(A)** List of species on the home page; **(B)** SSR statistics on the homepage; **(C)** Species page (*e.g.*, *Aegilops speltoides*); **(D)** Results page showing the desired search results; **(E)** Results page displaying SSR and corresponding primers; **(F)** Sequence BLAST tools; **(G)** *In silico* SSR prediction tools; **(H)** JBrowse framework.

To facilitate research on *Triticeae* crop breeding and improvement, we developed the *Triticeae* SSR database (TriticeaeSSRdb) accessible at http://triticeaessrdb.com/. This user-friendly web platform offers a range of features and charts for data visualization, browsing, and downloading. The web interface comprises five primary sections: Home, Species, Tools, Help, and Contact (**Figure 2**). Below, we provide a detailed description and instructions for utilizing the interactive pages within TriticeaeSSRdb.

#### 3.5.1 Home

The homepage of TriticeaeSSRdb provides an overview of the database and offers convenient access to plots and microsatellite data for *Triticeae* species. The homepage consists of two main sections. The first section displays a gallery of images containing SSR information for the 21 *Triticeae* species. Users can search for species using either their scientific or common names through the search bar or filter species of interest using the species panel. This comprehensive collection includes SSR details for all species, and the images are interactive, allowing users to click on specific species to access the second-level website interface dedicated to that species.

Additionally, the homepage features informative charts that summarize essential information about the database. These charts include the distribution of SSRs across species, the distribution of SSR motif types, the total number of SSRs, and the counts of SSRs located in genic and intergenic regions. These graphical representations provide a concise and visual representation of the database’s key characteristics.

#### 3.5.2 Species

The species selection panel, accessible from the top navigation bar, allows users to add or change species. This panel connects to the secondary website, which is the same as the species panel on the homepage. TriticeaeSSRdb provides species information, including a brief introduction, genome size, and total number of SSRs. A bar chart is available to visualize the distribution of SSR motif types and identify the most abundant motif category. Moreover, citation information for the reference genome is provided, enabling users to access relevant publications by clicking on the links.

The database offers versatile search options for retrieving SSRs based on various criteria, such as the genic or intergenic region, chromosome or scaffold-wise ID, motif type, repeat type, length, and genomic location. Notably, multiple chromosomes can be selected. In the advanced query, users can further refine their search by specifying parameters like the chromosome location (start and end coordinates) and size of SSR (length range). The result page dynamically displays tables with real-time visualization of the search results. The output includes comprehensive information that can be sorted by chromosome number, chromosome location, motif type, repeat number, and SSR size.

The search results provide a visualization of the repeat and designed primers in the flanking region, which is extracted from a sequence spanning 500 base pairs upstream (250 bp) and downstream (250 bp) of the repeat. Additionally, users can access three sets of designed primers for each SSR by clicking the view button. The primer information includes primer sequence, size, GC content, the melting temperature of forward and reverse primers, and product size. The primer page showcases the designed primers in the flanking region and allows users to download the information in text format using the download button.

#### 3.5.3 Tools

To enhance user experience, three practical tools have been integrated into TriticeaeSSRdb. The JBrowse framework, known for its versatility and customizability, is widely used for interpreting, and visualizing genomic features. For each species, specific gene annotation tracks have been established, including mRNA, CDS, UTR, and SSR loci, among other key data. This visualization plugin significantly improves the accessibility of genomic data, providing researchers with a powerful tool to access the desired genomic loci quickly and accurately, aiding in gene discovery and genetic breeding applications.

When users encounter sequence fragments without explicit IDs, TriticeaeSSRdb provides an online BLAST service that allows queries within the database collection of different *Triticeae* species. Users can submit amino acid or nucleotide sequences in Fasta format directly into the search box or upload text files. Depending on the query sequence and the database, users can select the appropriate BLAST algorithm to identify putative homologous sequences. In the advanced settings, users have the flexibility to set parameters such as expected threshold, Word size, and Max target sequences. After the alignment, the candidate hits are ranked in descending order of the e-value and displayed side by side in the result window.

We developed custom scripts to implement the MISA and Pytrf tools for the identification of SSRs in user input sequences. Users have the option to enter query sequences in the provided dialog box, and for larger file sizes, bulk identification can be performed by uploading files. Customization options allow users to specify the repetitive motifs for SSR detection. Once the search is initiated, the tool identifies microsatellites in the uploaded sequences and employs the primer3 program in the background to design primers for the target regions.

#### 3.5.4 Help

The Help page provides users with an introduction to the database and a comprehensive tutorial on how to effectively utilize its features.

#### 3.5.5 Download

To facilitate personalized local services for users, all raw data of this database are included under the Download interface. Users can select specific species according to their needs and download information such as whole-genome SSR identification results, SSR genomic distributions, and SSR annotations. Additionally, the statistical results in the supplementary tables can also be downloaded through this interface to enhance the user experience.

#### 3.5.6 Contact

The Contact page includes contact information for researchers to facilitate communication and provides quick access to generic external links to genomic resources. Additionally, the website offers researcher information and other relevant contact details.

## 4 Discussion

### 4.1 Characterization of SSR in *Triticeae* species

The application of SSR markers has demonstrated great value in genetic analysis, linkage mapping, breeding programs across different crop species, and germplasm characterization (Zhao et al., 2017). To further enhance the understanding of complex traits and facilitate molecular breeding, it is crucial to develop novel genome-wide markers and construct a physical map with uniformly distributed markers (Lu et al., 2019). Despite the growing popularity of single nucleotide polymorphism markers in *Triticeae* crop research, SSR markers continue to be widely utilized by research institutions due to their cost-effectiveness, simplicity, and rapidity (Li et al., 2015).

The advancement of improved sequencing technology and bioinformatics has resulted in the release of novel plant genome assemblies, particularly for cereal crops (Michael and VanBuren, 2020). This availability of genomic sequences for *Triticeae* species presents an opportunity to investigate and compare the distribution of microsatellites across these genomes. Computational approaches for SSR identification offer advantages such as high throughput, low cost, and minimal workload (Sharma et al., 2007). Various tools emerged from the late 20th century to the early 21st century to explore microsatellites within genome sequences. These tools include the Repeat Pattern Toolkit (RPT) (Agarwal, 1994), Tandem Repeats Finder (TRF) (Benson, 1999), Microsatellite Identification Tool (MISA) (Thiel et al., 2003), and Tandem Repeat Occurrence Locator (TROLL) (Castelo et al., 2002). However, the preference has shifted towards the utilization of microsatellite software that offers faster processing speeds, identification of SSR polymorphisms, and broader applicability. Notable examples of such tools include Krait (Pytrf) (Du et al., 2018) and CandiSSR (Xia et al., 2016).

In a previous study, we conducted a screening of genomic SSR loci in eight *Triticeae* species (Deng et al., 2016). However, existing genome databases have become outdated due to advances in sequencing technology and the release of new species genomes. Therefore, there is a need for comprehensive databases that collect, store, and maintain genomics data for further study of biological functions and molecular mechanisms. While some web resources provide information related to *Triticeae* species, they lack microsatellite markers, SSR prediction, PCR primer design, visualization, and other related information.

To address these limitations, we first characterized the distribution of SSRs in 21 *Triticeae* species using the most up-to-date genomes. In total, 3,891,705 microsatellites were identified. The distribution and frequency of SSRs vary among different species genomes. Our findings suggest that the relationship between SSR density and genome size is not evident in *Triticeae* species. Although differences in genome size may contribute to the level of microsatellite repetition, the density of SSRs has been found to be unrelated to genome size, consistent with previous studies (Behura and Severson, 2015; Portis et al., 2016).

Subsequently, we conducted a detailed evaluation of individual repeat motifs for each type of SSR in the *Triticeae* species. The base composition of SSR motifs in *Triticeae* species shows a strong bias toward A and T, consistent with their low GC content characteristics (Wang et al., 2022). Dimeric repeats were found to be the most abundant SSR motifs in *Triticeae* species. Previous studies have indicated that mono-nucleotide repeats are the most prevalent in *Poaceae* species. However, discrepancies in these findings may be attributed to the inherent limitations of next-generation sequencing methods used for data generation. In this study, we utilized the newly released reference genome, particularly integrating third-generation sequencing technologies, which greatly improved the accuracy of repeat sequence identification. Furthermore, we observed a significant increase in microsatellite abundance as the motif repeat number decreased, possibly due to higher mutation rates and increased instability in longer repeats.

### 4.2 TriticeaeSSRdb architecture, features, and advantages

The TriticeaeSSRdb, accessible at http://triticeaessrdb.com/, is a publicly available resource. It encompasses a comprehensive collection of 3,891,705 SSRs from 21 *Triticeae* species, with an overall frequency of 27.21 SSRs per Mb. These high-density SSRs serve as a valuable resource for genetic and genomic studies, including genetic diversity analysis, genetic mapping, marker-trait association, and molecular breeding. By facilitating the mining of microsatellite loci and the design of primers for genic and intergenic SSRs on a chromosome-wise basis, the database enables efficient genotyping with specific search criteria. Additionally, TriticeaeSSRdb incorporates additional tools such as genomic browsing, *in silico* SSR prediction for user-provided data, and BLAST-based similarity searches. TriticeaeSSRdb not only offers the largest collection of microsatellites but also provides a user-friendly web interface for data analysis and visualization. Through its statistical and plotting functions, users can easily grasp global microsatellite trends within a genome, eliminating the need for manual analysis of millions of data points. The information is presented through interactive plots and a filterable table view, ensuring that relevant microsatellite information is easily accessible to researchers.

TriticeaeSSRdb possesses specific advantages and features:

(i) TriticeaeSSRdb represents the pioneering effort in identifying and characterizing *Triticeae* SSRs, crucial for genetic and genomics research. It provides critical information on genetic variation, kinship relationships, gene mapping, and breeding. With a comprehensive collection of 3,891,705 SSRs across 21 *Triticeae* species, TriticeaeSSRdb stands as the most extensive and inclusive database for *Triticeae* SSR research.
(ii) TriticeaeSSRdb has been successfully developed as a one-stop, user-friendly platform for *Triticeae* species SSR research. *In silico* predicted markers can be easily searched based on criteria such as genic or genomic origin, chromosome-wise location, motif type, repeat type, length, and genomic position. TriticeaeSSRdb offers advanced search capabilities, including frequency-based searches and filtering based on motif type, repeat type, length, and genomic location. The search results provide visual representations of repeat motifs and flanking primers, with 500 base pairs upstream and downstream of the SSR. All results can be downloaded in clipboard, CSV, and Excel formats, facilitating further analysis. This genomic resource holds immense value for the global research community and can expedite chromosome-wise microsatellite locus mining and primer design for both genic and intergenic SSRs, enabling rapid genotyping.
(iii) TriticeaeSSRdb offers a range of practical tools, making it easy and efficient for users to browse, search, and download specific areas of interest. The database provides statistical analyses, including SSR distribution, motif categories, and repeat sequence composition. The platform supports data visualization in various formats. Additionally, TriticeaeSSRdb integrates external generic databases such as IPK, NCBI, e!DAL, and Ensembl Plants, enabling quick cross-database searches.

### 4.3 Prospects

TriticeaeSSRdb is continuously improving, and these resources contribute to understanding genomic research, species identification, and evolution in *Triticeae* species. In the future, we plan to enhance TriticeaeSSRdb with additional features and utilities:

(i) Continuous updates: As more *Triticeae* species datasets become available in the public domain, we will regularly update the database with newly released genomes, expanding beyond the *Triticeae* tribe and potentially encompassing the entire *Poaceae* family or even larger scales. With its scalable framework, TriticeaeSSRdb aims for at least one update per year.
(ii) Pan-genome integration: Recognizing that a single reference genome may not capture the full genetic information of a species, future research will shift towards pan-genomics, encompassing different subspecies, varieties, or strains with significant genetic variations. TriticeaeSSRdb intends to incorporate the pan-genomes of *T. aestivum*, *H. vulgare*, and other related species to enrich the genetic information available.
(iii) Integration of new analysis tools: TriticeaeSSRdb plans to integrate new analysis tools, especially those focused on pan-genomics and population genomics data, enabling cross-genome e-PCR and cross-validation to rapidly identify highly reliable and polymorphic SSR loci.
(iv) Integration of QTL information: We aim to enhance the database by incorporating QTL information derived from our studies as well as publicly available sources in our ongoing efforts. This integration will facilitate more accurate gene mapping and enable its application in breeding programs.

In summary, our approach maximizes the utility of TriticeaeSSRdb for studying various aspects of *Triticeae* crop development. It enables researchers, including those from small labs without extensive computing resources, to analyze genetic diversity and distinguish populations within *Triticeae* species.

## Data availability statement

The original contributions presented in the study are included in the article/**Supplementary Material**. Further inquiries can be directed to the corresponding authors.

## Author contributions

TL: Formal analysis, Visualization, Writing – original draft. SC: Data curation, Writing – original draft. ZC: Formal analysis, Visualization, Writing – original draft. YF: Resources, Writing – original draft. WL: Resources, Visualization, Writing – original draft. XZ: Supervision, Writing – review & editing. CL: Writing – review & editing. LC: Writing – original draft. WP: Writing – review & editing. YL: Supervision, Writing – review & editing.

## Supplementary material

Supplementary Figure 1 Distribution of SSR motif types with varying repeat numbers.

Supplementary Figure 2 Distribution overview of high-density SSR on chromosomes (using *T. aestivum* as a representative example). The bar depicts the number of SSR markers within a 1-Mb window.
