# Supplementary figures and images for: TriticeaeSSRdb: a comprehensive database of simple sequence repeats in Triticeae
Source: Front Plant Sci. 2024 May 22;15:1412953. doi: 10.3389/fpls.2024.1412953 (PMC11150838; doi:10.3389/fpls.2024.1412953)

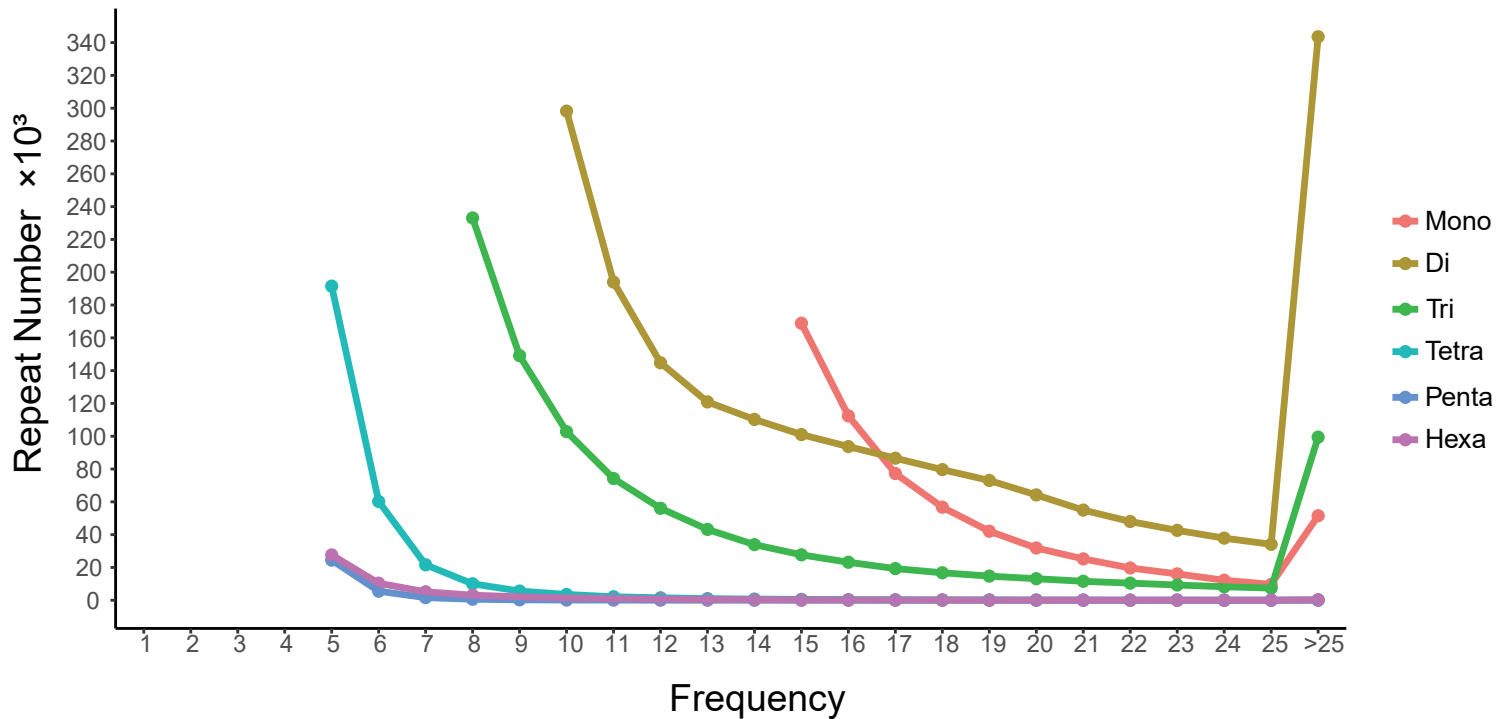

Supplement: Supplementary Figure 1 — Distribution of SSR motif types with varying repeat numbers. [file DataSheet_1.pdf]

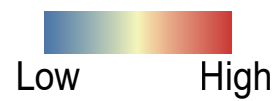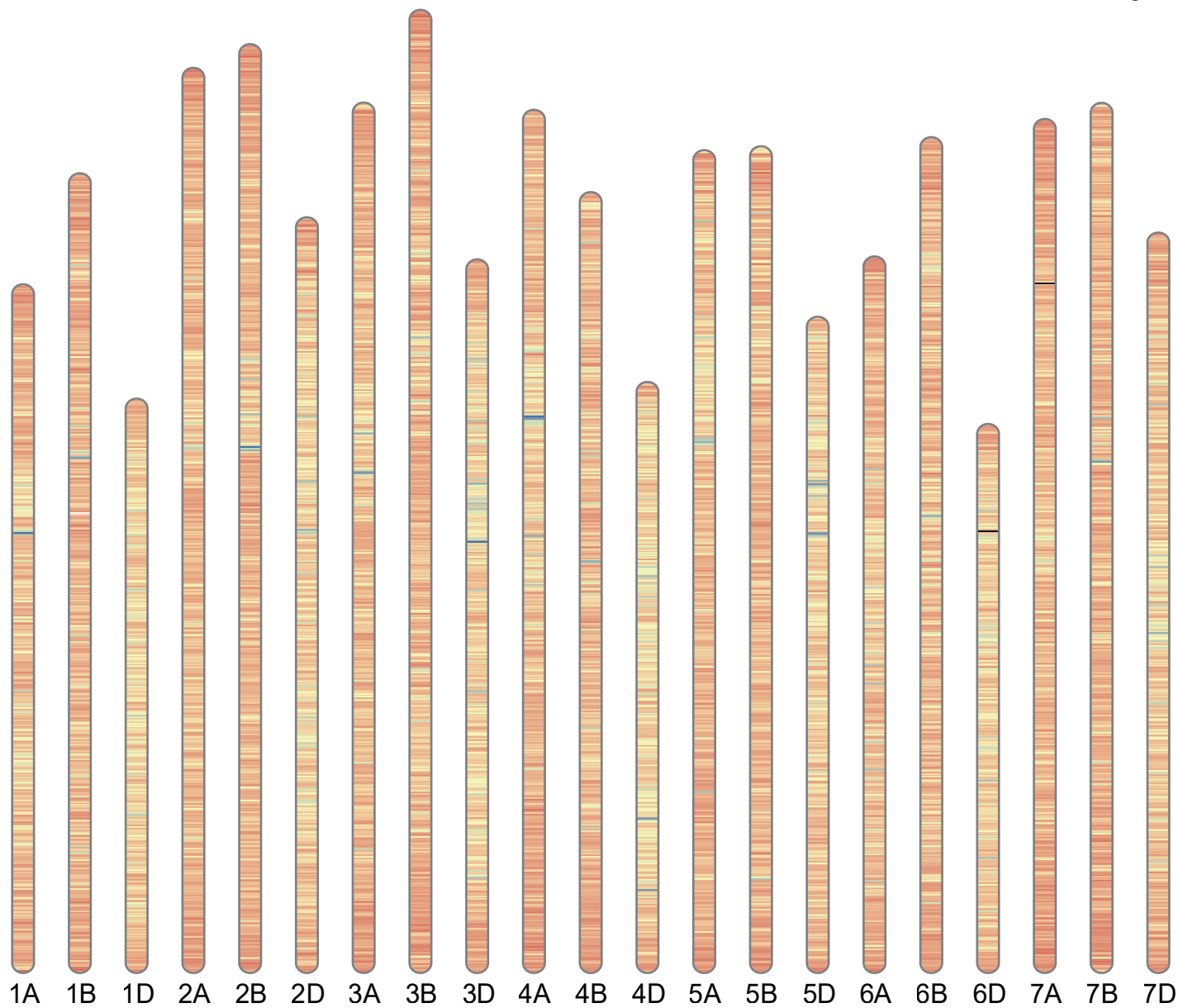

Supplement: Supplementary Figure 2 — Distribution overview of high-density SSR on chromosomes (using T. aestivum as a representative example). The bar depicts the number of SSR markers within a 1-Mb window. [file DataSheet_2.pdf]
